# Efficacy of ultra-micronized palmitoylethanolamide (um-PEA) in geriatric patients with chronic pain: study protocol for a series of *N*-of-1 randomized trials

**DOI:** 10.1186/s13063-016-1496-9

**Published:** 2016-07-29

**Authors:** Maura Marcucci, Federico Germini, Anna Coerezza, Luca Andreinetti, Lorenzo Bellintani, Alessandro Nobili, Paolo Dionigi Rossi, Daniela Mari

**Affiliations:** 1 Geriatric Unit, Fondazione IRCCS Ca’ Granda – Ospedale Maggiore Policlinico, Via Pace 9, 20122 Milan, Italy; 2 Department of Clinical Sciences and Community Health, Università degli Studi di Milano, via Francesco Sforza 35, 20122 Milan, Italy; 3 Laboratory for Quality Assessment of Geriatric Therapies and Services, IRCCS - Istituto di Ricerche Farmacologiche “Mario Negri”, via Giuseppe La Masa 19, 20156 Milan, Italy

**Keywords:** Aged, Analgesics, Chronic pain, Clinical trial, Elderly, Geriatric, *N*-of-1 trial, Normast, Palmitoylethanolamide

## Abstract

**Background:**

Chronic pain in older people is highly prevalent, often underestimated, and associated with adverse outcomes. Most available analgesic drugs are often either ineffective or not tolerated, with many side effects. Palmitoylethanolamide (PEA) is an endogenous widely distributed *N*-acylethanolamina involved in neuroinflammation and pain-generating processes. Formulations containing ultra-micronized palmitoylethanolamide (um-PEA) are available but their effectiveness on chronic pain in highly heterogeneous geriatric patients is not clear and probably not generalizable. We planned to adopt the *N*-of-1 trial approach to test the effectiveness of um-PEA objectively at the individual level in our older outpatients.

**Methods/Design:**

Persons 65 years or older referring to the Geriatric Unit of the Fondazione IRCCS Ca’ Granda Ospedale Maggiore Policlinico of Milan complaining of noncancer chronic pain of any origin will be eligible. Each trial will be a placebo-controlled randomized crossover trial including two um-PEA (600 mg twice a day) and placebo treatment pairs. The um-PEA or placebo 3-week periods will be separated by 2-week washout intervals to overcome possible carryover effects. Pain intensity, need of on-demand analgesic medications, and impact on daily activities will be evaluated. Cognitively impaired patients will be eligible as long as an expression of pain can be recognized and its frequency assessed by a caregiver. Trial results will be discussed with the patient or caregiver and the treating physician to decide whether to continue the treatment. The impact of the *N*-of-1 approach on the physician’s management plan and confidence will be assessed. We will secondarily meta-analyze the performed *N*-of-1 trials to obtain an estimate of the average effect of um-PEA compared with placebo using a frequentist and Bayesian approach.

**Discussion:**

While pursuing an ultimate clinical objective, i.e. to empirically and objectively decide the best treatment choice for an individual older patient with chronic pain, these series of geriatric *N*-of-1 trials on PEA will bring the principles of evidence-based medicine into the care of patients not usually represented in conventional randomized controlled trials, and realize a patient-centered outcome approach necessary to improve appropriate prescribing in elderly patients with multimorbidity and polypharmacy.

**Trial registration:**

ClinicalTrials.gov NCT02699281. Registered on 3 March 2016.

**Electronic supplementary material:**

The online version of this article (doi:10.1186/s13063-016-1496-9) contains supplementary material, which is available to authorized users.

## Background

Pain prevalence increases with age. In particular, the prevalence of chronic or persistent pain among older people ranges from 45 % to 80 % [1]. Persistent pain is a frequent reason for physician office visits among older persons [2], with women being more likely to report persistent pain than men [3]. Among older adults, the most frequent noncancer pain complaints are osteoarthritic back pain, especially in the low back or neck (around 65 %), musculoskeletal pain (around 40 %), peripheral neuropathic pain (typically due to diabetes or post-herpetic neuralgia, 35 %) and chronic joint pain (15 %–25 %) [4–6]. In fact, persistent pain may or may not be associated with a well-defined disease process [7].

Persistent pain and its inadequate treatment are associated with adverse outcomes in older people: functional impairment, falls, slow rehabilitation, mood changes, and sleep and appetite disturbances, resulting in a higher consumption of healthcare resources [8].

Achieving pain control in geriatric patients is complicated by many issues. First, older people may underreport pain, and in this population persistent pain has often complex and multifactorial manifestations. Self-reporting is deemed to be the gold standard in pain assessment but older people might find difficult to complete self-reported questionnaires of pain and functional status, developed and validated in younger patients [7, 9]. Pain evaluation is particularly cumbersome in older patients affected by dementia, who can express pain through unusual verbal and non-verbal modalities. Facial expression, verbalizations, non-verbal sounds, body movements, variations in interpersonal relationships, and mental status changes might be pain manifestations, and should be taken into account in pain evaluation even if extremely non-specific [1].

Second, the somatosensory system changes with aging [10], through the alteration or loss of myelinated and unmyelinated nerve fibres [11], an impairment in the endogenous opioid and non-opioid inhibitory systems of pain modulation [12], and a modified neuroplasticity [13]. All these changes lead to a reduced pain threshold, dysfunctional pain signaling, hyperalgesia, and central sensitization.

Third, pharmacological management of chronic pain is particularly challenging in the elderly. Owing to age-related body changes affecting drug pharmacokinetics, and because of multimorbidity and polypharmacy, older patients are generally more likely to experience drug-related adverse effects [7]. In particular, safety concerns can arise with most of the available analgesic drug classes. Given a better safety profile than traditional nonsteroidal anti-inflammatory drugs, acetaminophen is usually indicated as first line therapy for pain [7]. However, concerns about its efficacy on low back pain and osteoarthritis of the hip or knee, and about its actual safety, especially at the upper end of standard analgesic doses, have been raised by two recent systematic reviews of, respectively, randomized placebo-controlled trials [14] and observational studies [15]. Nonsteroidal anti-inflammatory drugs are more effective in the treatment of chronic inflammatory pain, but are burdened with many side effects, such as gastrointestinal bleeding, renal injury, and cardiovascular toxicity, which increase in frequency and severity with age [16, 17]. These safety issues are only partially mitigated with cyclooxygenase-2 selective inhibitors [18, 19]. Although their potential efficacy on persistent noncancer pain has been proved in some controlled trials, the adverse effects associated with opioids represent a barrier to their adoption and a frequent reason of drug discontinuation [20, 21]. In addition to other usual adverse effects, such as constipation and nausea, a negative impact on vigilance and cognitive performance is common in older persons. Newly prescribed opioid treatment has been associated with a higher risk of falls [22], so that the 2015 update of Beers criteria on potentially inappropriate medication use in older adults included it among the considerations on disease and syndrome interactions [23]. Although tolerance to central respiratory depression develops quickly, rapid dose increase, drug–drug interaction with other central nervous system depressants, and drug accumulation or accidental overdose are potential determinants of respiratory failure onset during opioid treatment [24].

So called adjuvant (or pain-modulating) drugs, co-administered with other analgesics, have been found to be effective in attenuating pain perception, particularly in the treatment of neuropathic pain [7]. They include antidepressants, anticonvulsants (e.g. pregabalin and gabapentin) and other medications that alter neural membrane potentials, ion channels, cell surface receptor sites, synaptic neurotransmitter levels, and other neuronal processes involved in pain signal processing. To minimize their potential adverse effects, these central nervous system drugs must be carefully titrated and monitored frequently [7].

All pain-modulating therapies target neurons, the principal component of the pain unit. However, a growing body of evidence suggests that immune cells, in particular mast cells and microglia, play a substantial role in the somatosensory system, being the primary interlocutors for pain neurons, both in the periphery and at the spinal and supraspinal levels [25]. Immune cells are located in proximity to sensory nerve endings and vasculature. After an injury or in the presence of an inflammatory stimulus, immune cells release mediators, such as bradykinin, prostaglandins, and histamine, stimulating nociceptors and playing an important role in the induction, amplification, and maintenance of chronic pain [26]. Physiological activation of microglia generally leads to resolution of neuroinflammation and restoration of tissue homeostasis. With aging, both microglia and mast cells increase their reactivity with a more intense response to a stimulus and a more robust production of pro-inflammatory cytokines lasting for an extended period [27, 28]. All these findings support the hypothesis that non-neuronal cells might be important therapeutic targets for the treatment of chronic pain, especially in older persons.

Palmitoylethanolamide (PEA) is an endogenous *N*-acylethanolamina widely distributed in different tissues [29]. It is synthesized on demand and its levels vary after stress or injuries, like those associated with pain [30]. In murine models of chronic inflammation and chronic or neuropathic pain, PEA seems to be able to reduce the recruitment and activation of mast cells, the production of pro-inflammatory mediators, and endoneural edema, thus reducing both pain and inflammation while preserving peripheral nerve morphology [31, 32]. Micronized or ultra-micronized PEA (m- and um-PEA) is available in Italy as an active molecule in several products classified as ‘Food for Special Medical Purposes’ (European Commission Directive 1999/21/EC), indicated for use ‘under medical supervision’ to treat conditions whose pathogenesis involves neuroinflammation. Evidence on the efficacy and safety of PEA in the treatment of chronic or neuropathic pain, alone or as add-on therapy, published as full articles or conference abstracts, in peer-reviewed or non-peer-reviewed journals, is currently based mostly on observational studies and case series, and on a few double-blind or open label randomized controlled trials [33–37]. Recently, in a meta-analysis of published and unpublished data, Paladini *et al*. [38] confirmed the efficacy of PEA on pain intensity independently of sex, age, and type of pain, even if with a smaller effect in people older than 65 years. Serious product-related adverse events were not reported in any of the included studies.

The mechanism of action and the safety profile make PEA an appealing choice for pain relief in older people. However, the evidence base for its efficacy to treat chronic pain in the elderly is not fully convincing. In addition, formulations containing um-PEA, like *Normast®*, are available in Italy only out-of-pocket and a treatment cycle at the doses recommended by the product information sheet would cost the patient about € 60. Given that the required treatment for persistent pain would probably be longer, the cost should be balanced by the actual effectiveness. These reasons represented the rationale to adopt a randomized *N*-of-1 trial as a more objective and personalized prescribing approach, compared with the conventional trial (i.e. ‘prescribe and see’) [39]. In general, *N*-of-1 trials are within-patient randomized multi-period crossover trials to compare, in a double-blind fashion, therapeutic strategies (e.g. an active drug versus no treatments, or two different active therapies), with time periods as randomization units and the patient as control for herself or himself. This study design can be theoretically used to test new drugs along their way to marketing authorization. In our case, we will adopt this approach to determine the best therapeutic choice for a certain patient in clinical practice, objectively and empirically, with randomization and blindness as instruments to overcome those factors (e.g. natural history of the disease, ‘placebo effect’, expectations) that might bias conventional trials of therapies [39–41]. In particular, the series of *N*-of-1 trials on PEA for chronic pain in older patients will be part of the activity of the Geriatric *N*-of-1 Service, an experimental project that we have implemented, with the approval of the local ethical committee, following the pioneering experience of Guyatt and colleagues [42] but in the specific context of geriatric medicine, which suffers most from the limits of parallel group randomized controlled trials, the current paradigms of evidence-based medicine [43].

## Methods/Design

This study protocol has been realized according to the CONSORT extension for reporting N-of-1 trials (CENT) 2015 [44] and the SPIRIT statements. The SPIRIT and CENT checklists for our paper are provided as Additional files 1 and 2, respectively.

### Study objectives

The primary (clinical) objective of our study is to apply the *N*-of-1 trial approach to test the effectiveness of um-PEA 600 mg (*Normast®*) twice a day for chronic pain in a certain patient referring to our geriatric unit in whom the treatment might be indicated, and to assist decisions on long-term treatment of that patient.

As a secondary (research) objective, we aim to obtain an overall estimate of the effectiveness of um-PEA 600 mg twice a day compared with placebo and evaluate the possible determinants of between-trial heterogeneity, through a meta-analysis of the *N*-of-1 trials performed for clinical purposes. Contextually, a classical frequentist meta-analytical approach will be compared with a Bayesian approach, and the potentialities of a Bayesian-based cumulative study design explored.

### Study design

**Fig. 1:**
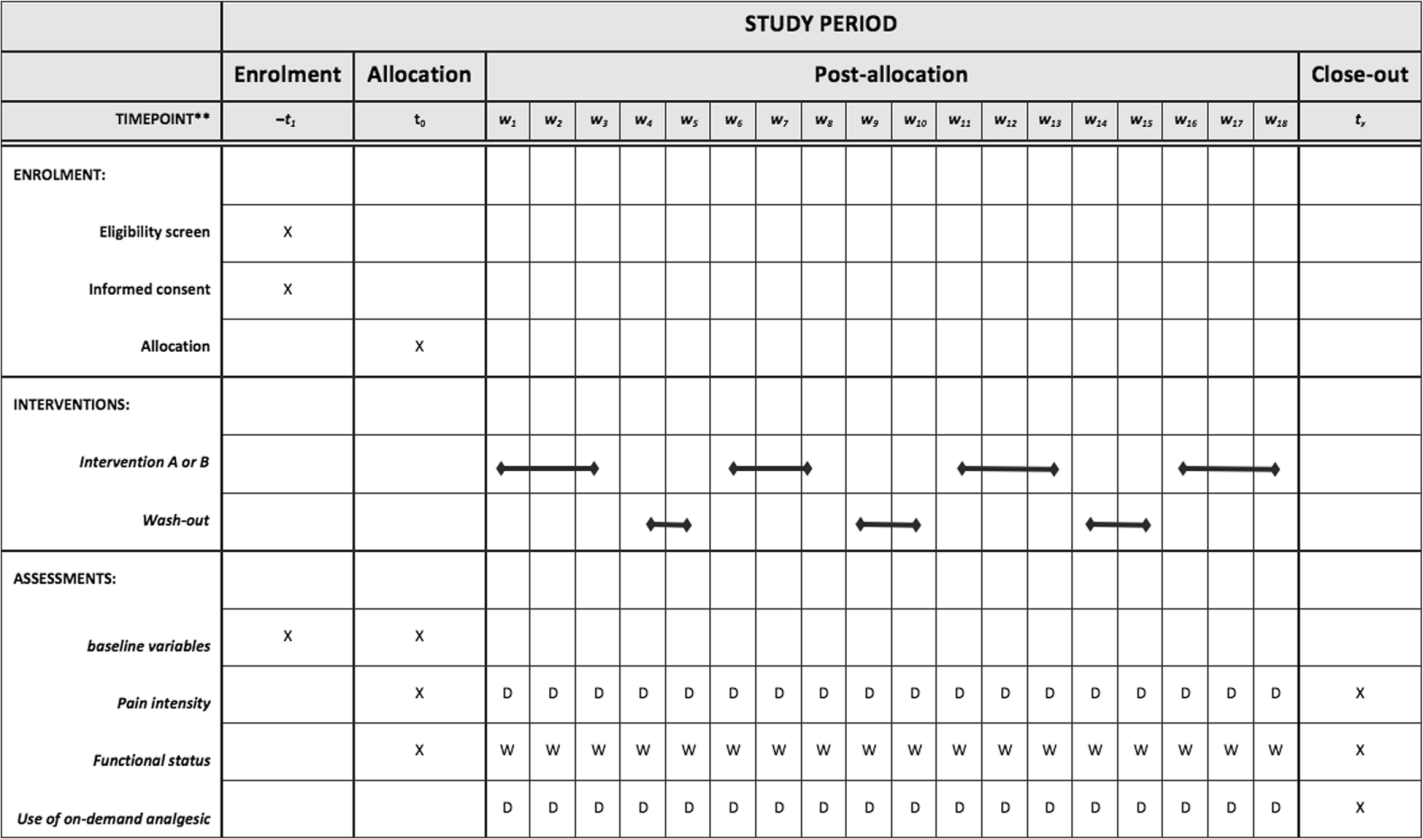
SPIRIT Study diagram. *W*_*n*_: week number, D: daily W: weekly. The study duration is 18 weeks. After the baseline assessment at time 0, patients will have two pairs of active drug (3 weeks) and placebo (3 weeks) exposures. Randomly assigned treatment pairs will comprise two treatment periods (active therapy or placebo) separated by a 2-week washout period. Treatment pairs will also be separated by washout periods

Each trial will be a blinded placebo-controlled randomized trial. Each trial’s duration will be 18 weeks, comprising two um-PEA and placebo treatment pairs assigned in a random order according to a pairwise randomization scheme. Thus, the sequence of each pair can be either um-PEA/placebo or placebo/um-PEA. Each treatment period will last 3 weeks. The treatment periods of each pair and the two pairs will be separated by 2-week washout intervals to minimize carryover effects. A run-in period will not be routinely performed, but will be considered when deemed appropriate (for example, in case of history of allergies to drugs or drug excipients). Figure 1 summarizes the schedule for the intervention and washout periods.

This study design was conceived taking into account the available information on the product pharmacological characteristics, balanced with the need for a trial with an acceptable duration from the practical viewpoint. The onset time of PEA is not easily predictable from its pharmacokinetics or pharmacodynamics. According to the available literature, most people notice the effects within 1 week, but sometimes 6–8 weeks are required, especially for chronic pain syndromes. Regarding a possible carryover effect, no sufficient data are currently available, either from basic or clinical research studies, to make a definitive statement. However, given the type of mechanism of action, a possible residual effect might be expected. Concerning the dose, in the heterogeneous literature on the use of the product for pain control, different doses have been tested, from 300 mg a day to 2400 mg a day, either once or split into three doses per day. The product information sheet for Normast® 600 mg suggests ‘1–2 tablets a day for 20–30 days’.

#### Eligibility criteria for participants

Older patients complaining of chronic pain of likely osteoarticular or neuropathic origin will be eligible for the study. Detailed *inclusion criteria* are:

- Patient age ≥ 65 years.
- Pain is localized at the back (any level) or at the joints or at the limbs.
- The pain is chronic, i.e. it has been present for at least 6 months, even if with fluctuations.
- The pain is attributable to one or more of the following conditions: osteoarthritis or osteoarthrosis; spondylosis; radiculopathy; diabetic peripheral neuropathy; post-herpetic neuralgia; chronic idiopathic axonal polyneuropathy; fibromyalgia; or pain of uncertain origin or idiopathic, as long as it has had and it is expected to have a chronic nature, even if with spontaneous fluctuations.
- The treating physician deems PEA to be a possible treatment option for the patient, either alone or as an add-on medication on top of other analgesic drugs.

Dealing with geriatric patients, and willing to offer the opportunity of a treatment option with an objective and empirical evaluation regardless of patient cognitive integrity, we will distinguish two cases:

- Case 1 (self-assessment): The patient is able to give an informed consent and to express the characteristics of the pain-like intensity and impact on function over a quantitative scale; the patient might be able to self-compile questionnaires, or to reliably answer questions administered by a caregiver.
- Case 2 (caregiver-based-assessment): The patient has a certain degree of cognitive decline that hinders the direct involvement of the patient in the consent process or in the outcome self-assessment, but an all-day caregiver willing to collaborate and consent to the trial is present. In this case, the pain has to be clearly expressed by the patient in a modality that can be assessed in its frequency and impact on function by someone other than the patient.

*N*-of-1 trials on case 1 patients will be candidates for the secondary objective of our study. The opportunity to meta-analyze case 2 trials will be judged according to the number and heterogeneity of the assessment instruments and modalities of completed trials.

Patients with cancer-related pain and patients with a clear ischemic pathogenesis for pain (e.g. intermittent claudication or critical limb ischemia) will be excluded. There will be no exclusion criteria concerning the comorbidities the patient might have. Patients can take other medications or undergo non-pharmacological therapies (e.g. physical therapies) for chronic pain control as long as they are not newly commenced; generally, no therapy modification that can affect pain must have been made soon before or concomitantly with the enrollment in the *N*-of-1 trial.

#### Setting and recruitment

The study will be conducted at the Geriatric Unit of the Fondazione IRCCS Ca’ Granda – Ospedale Maggiore Policlinico in Milan, Italy, which is an academic tertiary hospital unit that serves inpatient and outpatient referrals.

Patients will be mainly recruited from the geriatric outpatient clinic. Referrals might also be generated in the inpatient clinic but, to be enrolled in the study, patients need to have recovered from any acute illness.

### Interventions

One tablet containing either *Normast®* 600 mg or placebo will be administered orally twice daily during the treatment periods. No study drug will be administered during the washout period.

The patient will be allowed to take on-demand painkillers during the entire trial duration. At the baseline visit, the patient will be recommended according to our usual geriatric practice, i.e. to take acetaminophen at a maximum dose of 2 g daily if not contraindicated or in case of drug allergy or intolerance; if acetaminophen is insufficient, to combine or replace it with codeine or tramadol; to use ibuprofen when pain is not eventually controlled, trying to avoid, in general, nonsteroidal anti-inflammatory drugs. The patient will be given instructions on how to report on-demand analgesia according to the outcome assessment instruments and modalities.

### Outcome measures

Outcome measures evaluated in each *N*-of-1 trial will be the daily intensity of pain, the daily need of on-demand analgesic medications, and the impact of pain on daily activities measured over a week time (case 1: self-assessment trials).

In the cases in which self-assessment is not feasible (case 2: caregiver-based assessment trials) the daily frequency with which the patient complains of pain measured by the patient’s caregiver will be adopted as the outcome measure, since the measurement of the frequency of pain complaints by someone other than the patient is expected to be more feasible than the quantification of pain intensity. The daily need of on-demand analgesic medications and the impact of pain on daily activities over a week will also be evaluated in case 2 trials.

For the purpose of deciding for the optimal treatment in the patient, all the listed outcome measures will be valued. For meta-analytical purposes, the daily intensity of pain (case 1) or the daily frequency of pain symptoms (case 2) will be the *primary outcome measure*; the daily need of on-demand analgesic medications, and the impact of pain on daily activities will be the *secondary outcome measures*.

### Instruments, timing, and modalities of assessment

#### Case 1 (self-assessment)

**Fig. 2:**
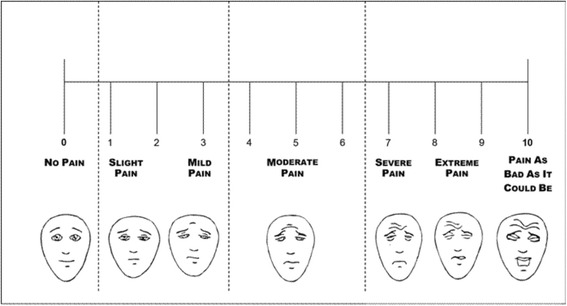
Pain assessment scale

**Fig. 3:**
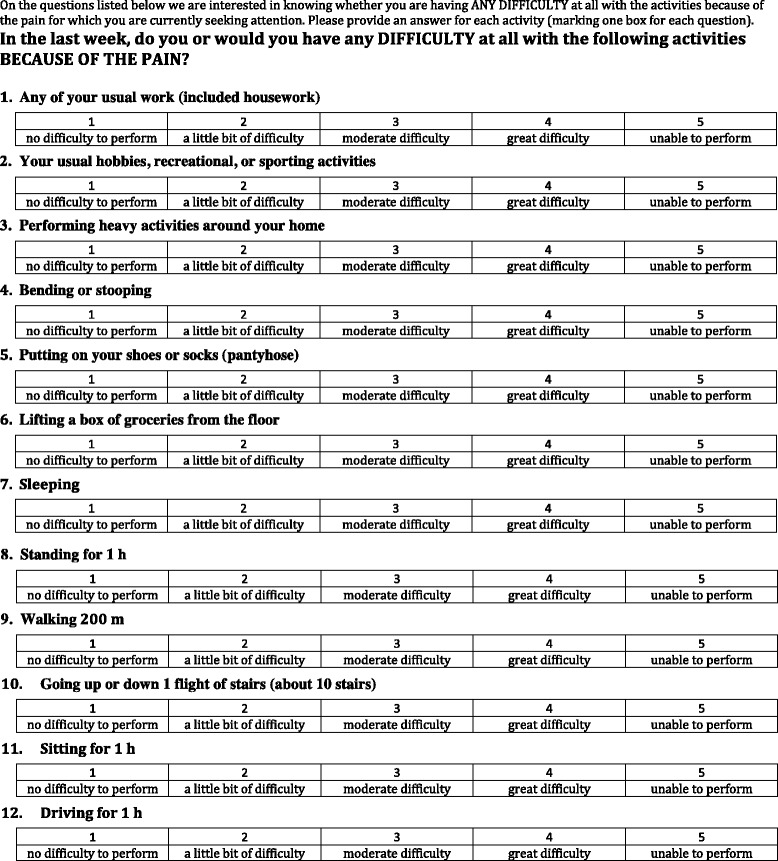
Questionnaire for functional status assessment

Pain intensity will be assessed daily using an 11-point (from 0 to 10) visual numeric scale [45]. To help with the assessment, in the paper used for the assessment, the numeric horizontal line will be accompanied with labels and pictures (modified from the Faces Pain Scale [46]) expressing the intensity of pain (Fig. 2). The patient or caregiver will also be asked to report the daily use of on-demand analgesic medications, specifying the type and dosage of drug taken (Fig. 2). The impact of pain on patient daily activities will be evaluated at the baseline and at the end of each week, using a short questionnaire modified from the Back Pain Functional Scale [47]. This is a self-report measure to evaluate the patient’s functional status, proposed for use in both clinical and research settings. It consists of 12 items (derived from existing questionnaires and interviews with physical therapists), investigating work, hobbies, home activities, bending or stooping, dressing shoes or socks, lifting, sleeping, standing, walking, climbing stairs, sitting, and driving. It has been validated [48] and extensively used [49–52], even with older people [53]. The scale was selected among the several available instruments for functional impairment due to chronic pain because of its comprehensiveness combined with a reasonable number of items. The questionnaire takes about 5 min to complete. Although it was conceived as a scale for back pain evaluation, the study authors also judged it as adequate to assess the functional impact of any osteoarticular or neuropathic pain. Although the scale has been used with older people, we slightly modified the Back Pain Functional Scale to make it more appropriate for geriatric patients. We reduced the walking distance from 1 mile (1610 m) to 200 m and the number of stairs from 20 to 10. In addition, we substituted the original rating scale (over six points) with a Likert five-point scale, in which 1 corresponds to ‘no difficulty to perform activity’, 2 to ‘a little bit of difficulty’, 3 to ‘medium difficulty’, 4 to ‘great difficulty’, and 5 to ‘impossibility to perform the activity’. The modified Back Pain Functional Scale is reported in Fig. 3. The total score is obtained as a mean of the scores for all items. When required, the questionnaire will be tailored to the patient usual activity and performance, thus removing those items that do not apply (e.g. if the patient does not drive, the question referring to driving will be removed). If at the enrollment visit the patient appears to value the impairment of one daily activity that is not included in the questionnaire, a specific item will be added.

At the baseline visit, the ability of the patient to compile the questionnaire unaided after instructions will be verified. If any limitation arises, the patient’s caregiver will be engaged and trained to take responsibility for completing the assessment.

#### Case 2 (caregiver-based assessment)

The participating caregiver and treating physician will be asked to identify the pain symptoms and to measure the daily frequency with which the patient complains of them using a five-point scale: 1 corresponds to ‘never’, 2 to ‘once’, 3 to ‘sometimes’, 4 to ‘often’, and 5 to ‘continuously’.

The impact on daily living will be measured by identifying those patient activities most affected by chronic pain (e.g. walking, sleeping, transfers) and building a personalized instrument through which the impact on each activity will be measured using a five-point scale similar to the one used for case 1 patients.

The use of on-demand analgesic medications will be reported daily by the participating caregiver, specifying the type of drug taken and its dosage.

#### Impact of N-of-1 trial on physician’s management plan

To assess the impact of the *N*-of-1 trial on the physician’s management plan, we will preliminarily ask each physician if he or she would treat the patient with um-PEA independently of the *N*-of-1 trial; once the *N*-of-1 trial results become available, we will ask how the physician intends to treat the patient. Management plan options will include continuing or withdrawing um-PEA. We will also investigate the level of the physician’s confidence in the management plan, both before and after the *N*-of-1 trial, using a seven-point scale. The physicians will be asked the following [42]:

How comfortable do you feel now about your treatment plan?

1. Totally comfortable; certain it is the right thing for the patient.
2. Almost totally comfortable; very probably it is the right thing for the patient.
3. Quite comfortable; it is probable that the treatment plan is best for the patient.
4. Not totally comfortable; but treatment likely to be as good as alternatives.
5. Mildly uncomfortable; some uncertainty whether treatment plan is best for the patient.
6. Moderately uncomfortable; feeling that the treatment plan might not be the best for the patient.
7. Extremely uncomfortable; uncertain about treatment plan and, if wrong, patient may suffer.

### Participant timeline

The study timeline is reported in Fig. 1.

At the enrollment visit, eligibility criteria will be checked, informed consent will be obtained, and data regarding patient socio-demographics, pain characteristics, comorbidities, and pharmacotherapy will be collected. The clinical investigators will also evaluate the ability of the patient and the caregiver to assess pain intensity, and appropriate training for questionnaire fulfilment will be performed.

After allocation, the study drug will be delivered to the patient (case 1) or caregiver (case 2) and baseline pain intensity and functional status will be recorded.

The patient or caregiver will be contacted by telephone after the first week of treatment to investigate possible issues concerning therapeutic adherence, adverse effects, and questionnaire completion. A visit with the patient and caregiver will be scheduled before the beginning of each period, to deliver the study drug and acquire the completed questionnaires, checking their correct fulfilment. At the end of the last period, the last questionnaires will be collected.

A final visit with the patient will be scheduled at the end of the trial, once the study results are available. The patient, the caregiver, the treating physician, and the study investigators will discuss the study results.

### Allocation and blinding

The sequence will be generated by the pharmacist using the web site www.randomizer.org. Two sets of two random numbers (numerals 1 [arbitrarily designated as placebo] or 2 [designated as active]), with each number in a set remaining unique, will be generated for each patient. Pharmacists will be recording the allocation sequence and will provide the study drug (either active drug or placebo, indistinguishable) to the clinical investigators at the beginning of each study period. Patients, participating caregivers, treating physicians, and clinical investigators (responsible for outcome assessment and data analysis) will be blinded to the treatment sequence.

Unblinding will be permissible at any time that the trial will be considered concluded. Reasons for which the trial will be deemed concluded other than its completion will be: the patient withdraws consent; the patient or the treating physician is convinced that drug effectiveness has been established or refuted before the end of the designed trial; and safety concerns.

### Sample size

For each *N*-of-1 trial, the sample size is represented by the number of periods of treatment. The choice to design each trial including two treatment pairs is not based on a formal power calculation, considering that *N*-of-1 trials with a practicable number of pairs can rarely reach the conventional level of statistical power of 80–90 % [54]. The choice is a trade-off between the desire of a trial as powerful as possible, and the practical need to limit its duration, given the required length of each period and of the washout between each treatment period. However, considering the actual objective of each *N*-of-1 trial, two treatment pairs seem a reasonable choice, given the starting good level of confidence on the treatment effectiveness that our geriatric team derived from the brief experience with the product (based on conventional trials) we preliminarily had in our practice.

A meta-analysis of the performed *N*-of-1 trials to estimate an overall population treatment effectiveness and evaluate the possible determinants of the effect heterogeneity between trials will be a proof-of-concept study. Given the explorative nature of this secondary objective, the number of *N*-of-1 trials that will be meta-analyzed is not dictated by a specific sample size calculation. A first meta-analysis will be performed on those completed *N*-of-1 trials initiated during the first 12 months from the start of the first *N*-of-1 trial, as long as there are at least three trials. A second meta-analysis will be performed including all the *N*-of-1 trials initiated within 24 months from the start of the first *N*-of-1 trial (study duration).

The posterior probability that um-PEA is better than placebo for a certain patient (single trial) and for the population (trial series) will be provided through a Bayesian approach.

### Statistical methods

According to the objectives of the study, we will first analyze results within each *N*-of-1 trial and, secondarily, we will combine results across them.

#### Analysis of individual N-of-1 trials

Results on daily pain intensity will be summarized, calculating first the mean symptom score of each week in each period, and then the mean score in each period. The week and period mean scores will be paired with the treatment administered in each period, in a cross-tabulation and in a graph. A similar synthesis will be used for the scores on the week impact on function. A paired *t* test will be used for the statistical comparison of period mean scores within treatment pairs.

At first, the analyses will be performed assuming that the washout periods were sufficient to overcome the possible carryover effect of PEA. If a slow onset time or a residual carryover effect are suspected from the qualitative (‘at a glance’) evaluation of the week (mean) outcome measures including those made during the washout periods, a sensitivity analysis will be conducted. In this second analysis, a paired *t* test will be repeated after having excluded from the calculation of the period mean scores the measures made during the first week of each period.

If more than three measures of the daily pain intensity in the same week are missing for at least one week, the period mean score will be computed, weighting each week mean score according to the inverse-variance method [55].

To make the final treatment decision as objective as possible, the results of each *N*-of-1 trial will first be evaluated by the physician and the patient or caregiver (when feasible), keeping the nature of the two treatments covered (i.e. naming the treatments simply A and B); then, the code will be broken and the nature of treatments revealed.

#### Meta-analysis of N-of-1 trial series

A linear mixed effect model [56] will be performed, with the week mean pain intensity score or the week impact-on-function score as dependent variable and the patient included as random effect (random intercept). The primary analyses will include treatment as the single explanatory variable with fixed effect. Secondly, a random coefficient for treatment (i.e. treatment effect varying between patients) will be tested. A random intercept for pair, in a nested structure (outcome measures within patients, and patients within pairs) will also be tested. A possible residual carryover effect (>2 weeks) will finally be tested, including sequence, pair, treatment by sequence interaction, and treatment by pair interaction as fixed-effects. The models will be also repeated excluding the first week measures of each period.

Patient-level (or study-level) variables will also be tested in the analyses as possible explanatory variables of treatment effect heterogeneity.

In addition to the classic (frequentist) analysis, results of the individual trials and their combination will be approached using Bayesian hierarchical methods, as described by Zucker et al. [57]. Non-informative priors will initially be modeled. In addition, physician’s pre-trial confidence in treatment effectiveness will be used to model informative priors.

#### Evaluation of N-of-1 trials on PEA

To evaluate the usage and usefulness of the performed *N*-of-1 trials, each trial will be classified according to:

- its *completeness*, defining as *complete* those trials conducted to the term; as *incomplete with clinical decision* those trials interrupted before the completion of the planned treatment pairs because the physician and patient or caregiver were at that point convinced of the effectiveness or lack of effectiveness of PEA (notwithstanding the blinding); and as *incomplete for other reasons* those trials interrupted before the completion of the planned treatment pairs because of other reasons (patient compliance, consent withdrawn, concurrent illness, death, etc.);
- the *achievement of statistically significant results*, setting *P* ≤ 0.1 as the criterion to define a statistically significant mean effect difference between PEA and placebo;
- the *direction of results*, i.e. a beneficial or harmful effect of PEA compared with placebo when a statistically significant mean effect difference is found.

### Monitoring

Owing to the small amount of data to be managed and to the absence of funding, a data monitoring committee is not recruited. Data will be managed and monitored directly by the study investigators. The correct use of the study drug and instruments, and possible adverse effects will be monitored at each phone or in person contact with the patient. Patients will be encouraged to report any possible adverse effect or concern about the study for the whole study duration through the provided investigators’ phone contacts.

### Data management

Data will be stored in an electronic database, protected by password.

## Discussion

Um-PEA might represent an effective and safe option for older patients with chronic pain. We opted to investigate this hypothesis at the individual level, in our routine practice, applying the *N*-of-1 approach to a geriatric population. Research and clinical practice become intimately intertwined on the ground of geriatric *N*-of-1 trials*.* Indeed, while they borrow typical research instruments (e.g. randomization, placebo) to achieve their ultimate clinical objective, i.e. to determine the best therapeutic choice for patients, reliably and empirically, they also meet the aims of innovative and timely research. They bring principles of evidence-based medicine into the care of older patients scarcely represented in conventional randomized controlled trials. Moreover, *N*-of-1 trials promote and exploit a patient-centered research that goes beyond the need of generalizability and even beyond the limits of the comparative effectiveness research. In particular, they embody the spirit of patient-centered outcomes research necessary to prioritize medicaments and improve appropriate prescribing in older patients with multimorbidity and polypharmacy [58]. Patient-centered outcomes research is indeed possible even with older patients who, contrary to expectations, have been shown to take into account competing outcomes when deciding on therapeutic alternatives, and to value the effects of treatments on cognitive, physical, and emotional functions [59].

We also expect that putting the patient at the center of our practice and research, modifying the trial design according to patient expectations, needs, and difficulties, might help to mitigate those well-known barriers concerning participant enrollment and adherence encountered in the conduction of trials involving older persons. Learning how to listen to the patient’s needs, even when not traditionally expressed, makes patient-centered practice and research possible even with cognitively impaired people. In particular, this series of *N*-of-1 trials on PEA will help to enhance our capabilities to interpret, measure, and treat pain in its alternative expressions.

The meta-analysis of the *N*-of-1 trials on PEA we conduct will provide aggregate information on the product that can be used to inform treatment decisions for other patients not participating in the trials.

Finally, the project, in its entirety, will represent a way of filling the gap between science and practice, facilitating the involvement of patients and clinicians in the production of evidence, and promoting the creation of ‘a real clinical learning community’ [60].

## Trial status

At the time of manuscript submission, four patients have been enrolled and randomization has begun.

## Abbreviations

CONSORT, Consolidated Standards of Reporting Trials; PEA, palmitoylethanolamide; um-PEA, ultra-micronized palmitoylethanolamide; SPIRIT, Standard Protocol Items: Recommendations for Interventional Trials

## Additional file

Additional file 1: SPIRIT 2013 checklist. (DOC 120 kb) Additional file 2: CENT 2015 checklist*; CONSORT 2010 checklist items with modifications or additions for individual or series of N-of-1 trials; empty items in the CENT 2015 column indicate no modification from the CONSORT 2010 item. (DOCX 97.2 kb)
